# Linking Ambidextrous Organizational Culture to Innovative Behavior: A Moderated Mediation Model of Psychological Empowerment and Transformational Leadership

**DOI:** 10.3389/fpsyg.2019.02192

**Published:** 2019-10-11

**Authors:** Yanbin Liu, Wei Wang, Dusheng Chen

**Affiliations:** ^1^School of Business, Ningbo Institute of Technology, Zhejiang University, Ningbo, China; ^2^Business School, Ningbo University, Ningbo, China; ^3^Hikvision Digital Technology Co. Ltd, Hangzhou, China

**Keywords:** ambidextrous organizational culture, innovative behavior, psychological empowerment, transformational leadership, self-determination theory

## Abstract

Research into innovative behavior is not new, but its importance for organizational effectiveness has become even more evident in recent years. However, the psychological processes and underlying mechanism concerning how and why innovative behavior occurs within an organization still invite more investigation. The present study considers ambidextrous organizational culture as a pro-innovation culture and proposes that it can be perceived by employees, which leads to their innovative behavior. This study adds clarity by exploring the impact of perceived ambidextrous organizational culture on employees’ reactions related to innovation *via* the intermediate mechanism of psychological empowerment and the moderating condition of transformational leadership. Hypotheses are derived from a motivational perspective based on self-determination theory. Results are based on data collected from 647 Chinese employee-supervisor dyads. This study finds that employees’ perceptions of ambidextrous organizational culture have an indirect effect on innovative behavior through psychological empowerment. Specifically, the positive indirect relationship is amplified when transformational leadership is at a higher level. Our findings show how the mediating mechanism of psychological empowerment and the moderating condition of transformational leadership work together to improve innovation by individuals. The findings reveal several ways in which organizations can strategically focus on their cultural and supervisory training, such as applying this model to improve employees’ outcome related to innovation.

## Introduction

Ambidexterity, referring to the organizational ability of exploring new capabilities and exploit existing competences simultaneously (Gupta et al., 2006), has been described as an important antecedent of organizational innovation (Gibson and Birkinshaw, 2004; Cao et al., 2009; Junni et al., 2013). Considering that individual innovation is the foundation of organizational innovation (Amabile, 1988), researchers have argued that the relationship between ambidexterity and individual innovation should not be ignored (Rosing and Zacher, 2016). As is widely accepted, innovative behavior as individual innovation is of vital importance for organizational effectiveness (e.g., Janssen et al., 2004; Yuan and Woodman, 2010). Innovative behavior refers to an employee’s intentional adoption or adhibition of new ideas, products, processes, and procedures which are work-related (West and Farr, 1989). Scholars argue that innovative behavior by employees, such as initiating and implementing new ideas related to work and products, plays a critical role in organizational success (West and Farr, 1990). Meanwhile, there are still several crucial questions about the concept of ambidexterity that have not been sufficiently answered: first, exploration and exploitation are taken as paradoxical; second, little is known about how to integrate exploration and exploitation to pursue innovation (Wang and Rafiq, 2014).

Gibson and Birkinshaw (2004) argue that ambidexterity is embedded in the type of organizational culture which promotes creativity and discipline at the same time and thus can help integrate exploration and exploitation to facilitate innovation. Wang and Rafiq (2014) conceptualize this type of organizational culture as ambidextrous organizational culture for the first time. It consists of two dimensions: organizational diversity and shared vision. The former is defined as “a set of organizational values and norms that encourage and tolerate differences, and recognize and reward individuals’ different viewpoints, skills and knowledge” and the latter is defined as “a set of organizational values and norms that promote the overall active involvement of organizational members in the development, communication, dissemination, and implementation of organizational goals” (Wang and Rafiq, 2014, p. 62). Organizational diversity encourages employees to engage in actions required for exploration (Ahuja and Lampert, 2001) while shared vision converts diversity into focused ideas and actions required for exploitation (Leana and Van Buren, 1999), and under this type of culture exploration and exploitation are integrated together. Empirical evidence also suggests that organizational diversity and shared vision as components of ambidextrous organizational culture help integrate exploration and exploitation required by contextual ambidexterity and consequently influence new product innovation (Wang and Rafiq, 2014).

However, the relationship between ambidextrous organizational culture and innovative behavior is still unclear, especially its underlying mechanism and conditional limits. Although Wang and Rafiq (2014) provide a path from ambidextrous organizational culture to product innovation *via* contextual ambidexterity, this path follows a resource-based perspective lacking of consideration of employees’ psychological needs and motivation that are important for individual innovation (Zhang et al., 2015). The present study draws on a motivational perspective and takes employees’ self-determination into account, exploring how employees perceive ambidextrous organizational culture and react by innovative behavior.

We use ambidextrous organizational culture as the antecedent variable for two reasons: (1) theoretically, this concept reflects the nature of organizational culture by manifesting its dual aspects of external adaptation (change and flexibility) and internal integration (stability and direction) (Denison and Mishra, 1995; Schein, 2004) and thus can help integrate exploration and exploitation required by ambidexterity that promotes innovation (Wang and Rafiq, 2014); (2) practically, it emphasizes the involvement and participation of employees as part of the organizational culture, compared with traditional top-down types of culture (Ghoshal and Bartlett, 1994; Gibson and Birkinshaw, 2004; Wang and Rafiq, 2014), and revealing its relationship with innovative behavior can help attract practitioners’ attention to build organizational culture with a bottom-up approach.

Drawing on the theory of self-determination, we use psychological empowerment as the mediator and transformational leadership as the moderator in our hypothesized model. When employees perceive a higher level of ambidextrous organizational culture, their psychological needs are more easily to be fulfilled and thus get psychologically empowered (Randolph, 1995; Seibert et al., 2011). Employees are motivated to act based on their intrinsic psychological needs such as autonomy, competence, and relatedness (Deci and Ryan, 2002), indicating that employees who feel more psychologically empowered are more likely to overcome motivational difficulties during the process of innovation (Schermuly et al., 2013). So far, we have proposed psychological empowerment as a mediator. Moreover, we argue that transformational leadership can amplify the positive linkage between ambidextrous organizational culture and innovative behavior through psychological empowerment. Since the path from ambidextrous organizational culture to innovative behavior *via* psychological empowerment is a process of employees’ psychological needs being fulfilled and intrinsic motivation being activated, and transformational leaders help to confirm and strengthen employees’ perceptions of the culture by providing them individualized consideration and shared goals (Shalley, 1995; Duan and Huang, 2014; Qu et al., 2015), we argue that transformational leadership facilitate the effect of perceived ambidextrous organizational culture on psychological empowerment.

In summary, we argue that ambidextrous organizational culture can appear as a pro-innovation culture and be perceived by employees and thus lead to their innovative behaviors. To further probe into its underlying mechanism, we propose a first-stage moderated mediation model where employees’ psychological empowerment plays a mediating role while transformational leadership serves as a moderator. The intended contributions of this study to the existing literature are threefold. First, this study focuses on ambidextrous organizational culture where contextual ambidexterity is grounded and connects this type of culture to individual innovation by exploring employees’ perceptions and reactions at a micro-level perspective. As a critical factor of organizational context (Denison, 1996), organizational culture is traditionally considered as a higher order construct and shaped by a top-down approach (Wang and Rafiq, 2014). This study probes into the concept of ambidextrous organizational culture which is formed with a bottom-up process and follows a motivational perspective that links employees’ perceptions of the culture and their innovative behavior together, shedding some light on the psychology of organizational culture and innovation by exploring employees’ perceptions and reactions under an ambidextrous organizational context. Second, this study extends the scope of self-determination theory by linking ambidextrous organizational culture and innovative behavior through psychological empowerment. Previous studies link ambidexterity and innovation mostly based on a resource-based perspective (Wang and Rafiq, 2014). The present study finds that psychological empowerment is shaped by employees’ perceptions of ambidextrous organizational culture and in turn results in different levels of innovative behavior, which reflects a mechanism of employees’ psychological needs being fulfilled and intrinsic motivation being activated and consequently innovative behavior being exhibited. Third, this study reveals that the interaction of the perceptions of ambidextrous organizational culture and transformational leadership leads to a higher level of psychological empowerment, and then a higher level of innovative behavior, which supports examinations of impacts of organizational culture and leadership on employee outcomes in an integrated way rather than separately.

### Ambidextrous Organizational Culture and Innovative Behavior

Organizational ambidexterity as a metaphor referring to a company’s ability to explore new competences and exploit existing competences at the same time has attracted interest especially in innovation research (Gibson and Birkinshaw, 2004; Simsek et al., 2009). Traditional views take exploration and exploitation as competing organizational activities (Duncan, 1976), indicating that they are structurally and temporally separated to achieve balance (Gupta et al., 2006). Recent research suggests that ambidexterity is not only an organizational level construct but can also be operationalized at individual and team levels (Birkinshaw and Gupta, 2013). Scholars conceptualize individual ambidexterity of managers and define managers’ explorative behavior as “searching for, discovering, creating, and experimenting with new opportunities” and exploitative behavior as “selecting, implementing, improving, and refining existing certainties” (Mom et al., 2007, p. 910). Rosing and Zacher (2016) find the positive relationship between individual ambidexterity and innovative performance, and emphasize the balance of exploration and exploitation. Unlike considering exploration and exploitation as a bi-polar construct, Gibson and Birkinshaw (2004) suggest the possibility and necessity of contextual ambidexterity referring to simultaneous exploration and exploitation within a business unit. Contextual ambidexterity emphasizes the integration of exploration and exploitation within a business unit and allows firms to both succeed in the short term and achieve long-term sustainability (Gibson and Birkinshaw, 2004; Simsek et al., 2009).

Contextual ambidexterity is considered to be grounded in the type of organizational culture (Ghoshal and Bartlett, 1994; Gibson and Birkinshaw, 2004), which can accelerate creativity and discipline (Jelinek and Schoonhoven, 1993). Combining insights from organizational identity and organizational learning, Wang and Rafiq (2014) conceptualize ambidextrous organizational culture and find its positive relationship with contextual ambidexterity. They argue that within an organization, if employees hold different knowledge and skills that facilitate creativity (organizational diversity), assuming these differences reflect their shared goals and norms that promote discipline (shared vision), an ambidextrous organizational culture will form (Wang and Rafiq, 2014).

This study takes employees’ perceptions into consideration instead of exploring ambidextrous organizational culture in higher order, for the reason that innovative behavior as a consequence is an individual-level outcome, and is taken as employees’ reactions based on their perceptions, supporting the viewpoint that individuals tend to “react on the bases of perceptions of reality, not reality *per se*” (Ferris and Judge, 1991, p. 464). Moreover, this study takes ambidextrous organizational culture as a pro-innovation culture instead of other types of organizational culture for the reason that this culture is developed by a bottom-up process rather than the traditional top-down approach, which emphasizes individual involvement and participation (Wang and Rafiq, 2014). Since innovative behavior reflects employees’ intentional behavior related to innovation in the workplace (West and Farr, 1990), organizational culture that can get employees involved and stimulate their intrinsic motivation would promote the likelihood of innovative behavior occurring. Accordingly, drawing on self-determination theory, we build the path from ambidextrous organizational culture to innovative behavior based on a motivational perspective.

### The Mediating Role of Psychological Empowerment

Following the psychological perspective, psychological empowerment refers to a psychological state that reflects four aspects of cognition toward an employee’s work role: meaning, competence, self-determination, and impact (Spreitzer, 1995). Meaning involves a fit between a work role’s requirements and values and beliefs of an employee (Brief and Nord, 1990; Spreitzer, 1995). Competence reflects an employee’s feelings of self-efficacy that one is capable to perform a task successfully (Bandura, 1989). Self-determination refers to an employee’s sense of autonomy in making choices and regulating actions (Deci et al., 1989). Impact refers to the degree to which an employee believes he or she can make a difference in organizational outcomes (Spreitzer, 1995).

An empowerment perspective emphasizes that circumstances that surround employees are important facilitators to psychological empowerment for the reason that psychological empowerment is a set of cognitive factors shaped by work environments and contexts (Thomas and Velthouse, 1990). And organizational culture is thought to be one of the critical contextual factors (Wang and Rafiq, 2014). Under the conditions of ambidextrous organizational culture, employees are encouraged to respect different knowledge, skills, and abilities; and based on this openness to differences, they build shared values, norms, and goals. When employees understand the vision and goals of the collective, and have feelings of the importance of openness and teamwork emphasized by the organization, they are more likely to take empowered actions (Siegall and Gardner, 2000). We find that ambidextrous organizational culture is a critical contextual factor that positively relates to employees’ psychological empowerment.

We follow self-determination theory (Deci and Ryan, 2000) to clarify the mediating role of psychological empowerment. Under the circumstances that employees perceive a higher level of ambidextrous organizational culture, diverse individual knowledge, skills, and abilities promoting creativity reflect shared expectations and group norms (Rink and Ellemers, 2007), and employees’ needs of autonomy, competence, and relatedness are more likely to be fulfilled, indicating that they are more psychologically empowered (Seibert et al., 2011; Fernandez and Moldogaziev, 2015). More specifically, organizational diversity reflects the values that encourage employees to be more opening to task-related differences (Rink and Ellemers, 2007). It can be taken as support for tolerating differences in viewpoints, skills, and knowledge (Ferner et al., 2005), which provides employees with feelings of being accepted by the organization and a sense of self-determination (Seibert et al., 2011). Employees’ perceiving shared vision is a bottom-up process, and this process includes transferring knowledge and sharing information (Wang and Rafiq, 2014), which are keys to building psychological empowerment (Randolph, 1995).

Psychological empowerment enhances “the ability of employees to implement their ideas and suggestions for change, resulting in greater innovation at work” (Seibert et al., 2011, p. 986). Based on a self-determination perspective, since employees’ basic needs of autonomy, competence and relatedness are satisfied, psychologically empowered employees obtain a higher level of intrinsic motivation (Ryan and Deci, 2000), and thus are more likely to put forth new ideas and execute incremental innovation (Singh and Sarkar, 2012). Evidence also shows that self-determined and impactful employees are more likely to test new ideas (Schermuly et al., 2013), employees who believe in their competence are more creative (Zhou, 1998), and employees with meaningful commitment in their tasks has also been demonstrated to be associated with innovative behavior (Bass, 1985; Singh and Sarkar, 2012). Accordingly, we propose that psychological empowerment mediates the relationship between employees’ perceptions of ambidextrous organizational culture and their innovative behavior.

Hypothesis 1: Employees’ psychological empowerment mediates the relationship between perceived ambidextrous organizational culture and innovative behavior.

### The Moderating Role of Transformational Leadership

As mentioned above, the path from employee perceiving ambidextrous organizational culture to innovative behavior *via* psychological empowerment is a process of psychological needs (i.e., autonomy, competence, and relatedness) being fulfilled and intrinsic motivation being activated. We further propose that there are moderating conditions of this path. Considering that supervisors have a large impact on their subordinates’ feelings and cognition (Dienesch and Liden, 1986), and subordinates’ perceptions of their work roles and experiences are shaped by their leaders in the workplace (Liden et al., 1997), it is contended that supervisors are important providers of empowering experiences to their subordinates (Deci et al., 1989). Thus, the results might be different if leadership changes.

Transformational leaders motivate and encourage their subordinates to perform beyond their expectations (Podsakoff et al., 1990) and promote employees to form autonomous motivation by fulfilling their psychological needs related to self-determination (Bass, 1985; Kovjanic et al., 2012; Duan and Huang, 2014). Subsequently, transformational leadership can be a facilitator to the process of employees perceiving ambidextrous organizational culture and getting psychologically empowered, for the reason that employees’ needs are more easily to be satisfied and thus they get more empowered when their supervisors exhibit a higher level of transformational leadership. Therefore, we choose transformational leadership as a moderator in the present study and propose that:

Hypothesis 2: Transformational leadership positively moderates the relationship between perceived ambidextrous culture and psychological empowerment.

Following self-determination theory, contextual factors within an organization (e.g., ambidextrous organizational culture) that can meet employees’ psychological needs promote their intrinsic motivation (Bass, 1985), which drives employees to be willing to engage in innovation-related work (Zhang et al., 2015). During this process, transformational leaders can provide individualized consideration and shared goals for employees (Shalley, 1995; Duan and Huang, 2014; Qu et al., 2015), which confirm and strengthen employees’ perceptions of ambidextrous organizational culture by fulfilling the psychological needs of employees (Bass, 1985; Kovjanic et al., 2012; Duan and Huang, 2014). It is contended that the relationship between perceived ambidextrous organizational culture and psychological empowerment is stronger when employees’ supervisors are transformational, for the reason that employees’ perceptions of ambidextrous organizational culture are reinforced by transformational leadership. Moreover, stronger psychological empowerment amplified by the interaction of ambidextrous organizational culture and transformational leadership indicates stronger intrinsic motivation, leading to a higher level of innovative behavior.

Hence, we propose transformational leadership as an enhancer to the first stage of the positive path from perceived AOC to innovative behavior through psychological empowerment.

Hypothesis 3: Transformational leadership positively moderates the first stage indirect effect through psychological empowerment, and the indirect effect is stronger when transformational leadership is high than when it is low.

**Figure 1:**
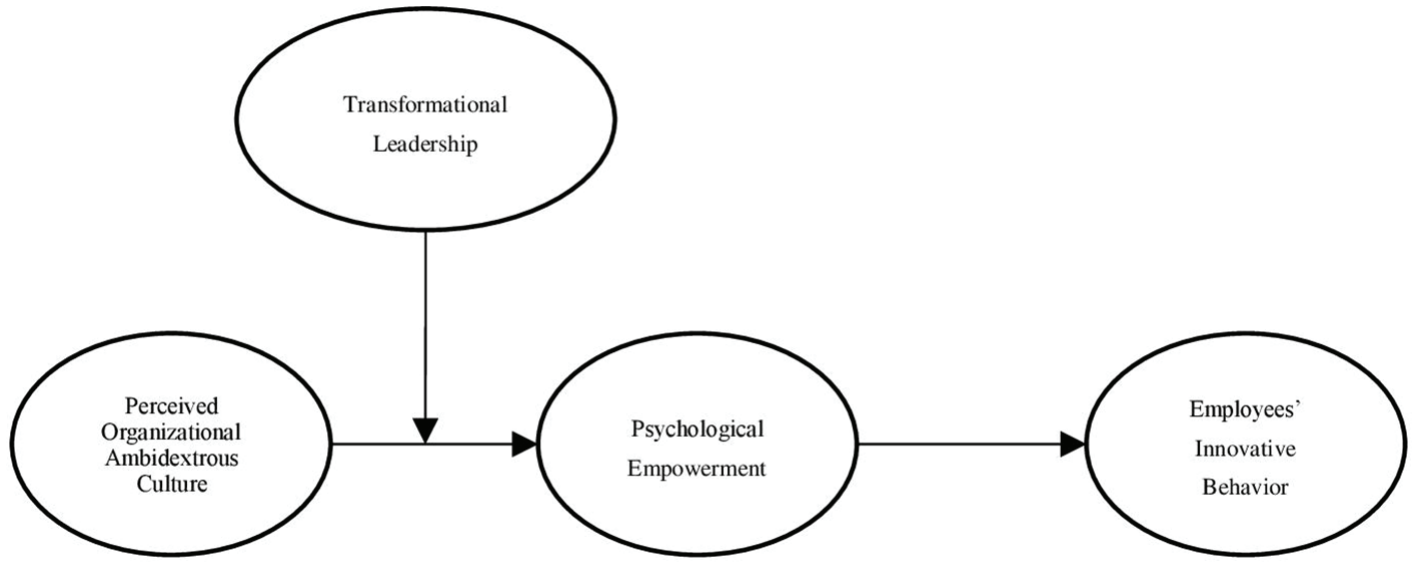
The moderated mediation model.

Based on these hypotheses, we propose a new moderated mediation model that outlines the relationship between perceived ambidextrous organizational culture and innovative behavior and its underlying mechanism. The theoretical model is schematically represented in Figure 1.

## Materials and Methods

### Sample and Procedure

Participants were 647 employees from Transfar Group, a private enterprise group focused on the chemical industry. The group had more than 12,000 employees at the time of this study in 2016 and was in a critical phase of implementing organizational change to better cope with the dynamic business environment. The sample was highly diverse. Participants’ age ranged from 20 to 58 years (mean = 30.47, SD = 6.18), and 35.09% percent of them were female. Half of the participants held college degrees (51.62%). The sample is comprised of employees working in 47 different departments (administration: 11.75%; financial: 5.41%; purchasing: 10.51%; R&D: 16.69%; marketing: 18.08%; plant: 37.56%). The average employment tenure was 4.42 years (SD = 4.10).

Data were collected with the support of the HR department for 3 months (July 2016–September 2016), and surveys were conducted monthly. The first measure asked employees to assess their perceptions of ambidextrous organizational culture and their immediate supervisors’ transformational leadership in early July. Employees rated themselves on psychological empowerment in early August as the second measure. In the third-round data-collection a month later, employees’ innovative behaviors were individually evaluated by their immediate supervisors. Every business unit has its own department number while employees and supervisors have their own job numbers. They were asked to provide those numbers at the beginning of every round of the measurement. By matching these numbers, we can pair the subordinate-supervisor dyads.

Electronic copies of questionnaires were handed out to employees and their supervisors by email. In the first and second rounds, responses of 1,142 employees and 813 employees were obtained, respectively. In the final round, feedback from 695 employees and 110 supervisors was acquired. After deleting those unmatched pairs of supervisors and subordinates, a sample of 647 supervisor-subordinate dyads was received, with a final response rate of 56.65% by employees. We stressed confidentiality in every cover letter, informed consent form, and questionnaire, to state that all data collected was only for research purposes.

### Measures

The present study used three questionnaires. The first-round version contained demographic variables and items about perceived ambidextrous organizational culture and transformational leadership. The second-round version measured psychological empowerment, and the third-round survey measured innovative behavior.

All items were extracted from existing literature and adapted to fit this study. All measures were translated to Chinese following a procedure of standard translation-back-translation (Reynolds et al., 1993). All the items used Likert-type scales (1 = “strongly disagree” to 5 = “strongly agree”).

*Perceived ambidextrous organizational culture* was measured by seven items from Wang and Rafiq (2014). Employees were asked to evaluate how they perceive the organizational culture of interest described by each item. This measure includes two dimensions: organizational diversity, and shared vision. Organizational diversity is measured by three items, an example of a typical item being: “we respect everyone’s different viewpoints.” Shared vision is measured by four items, such as “all employees view themselves as partners in charting the direction of this company.” The Cronbach’s *α* was 0.92.

*Psychological empowerment* was measured using a 12-item scale from Spreitzer (1995). This measure includes four dimensions: meaning, competence, self-determination, and impact, each dimension containing three items. Samples of these items are “The work I do is very important to me,” “I am confident about my ability to do my job,” “I have significant autonomy in determining how I do my job,” and “My impact on what happens in my department is large,” respectively. The Cronbach’s *α* was 0.88.

*Innovative behavior* was measured by a 6-item scale from Scott and Bruce (1994). Supervisors were asked to rate how characteristic each of the listed behaviors was of a particular employee. Sample behaviors are “generates creative ideas” and “promotes and champions ideas to others.” The Cronbach’s *α* was 0.86.

*Transformational leadership* was measured by 20 items taken from including idealized influence, inspirational motivation, intellectual stimulation, and individualized consideration (Bass and Avolio, 1997). A sample item is “provides reasons to change my way of thinking about problems.” The Cronbach’s *α* was 0.85.

*Control variables* included employees’ age and company tenure. Previous research indicates that these two variables could influence individuals’ innovation-related behavior (see Ng and Feldman, 2013).

### Analytic Strategy

A confirmatory factor analysis was conducted using AMOS 22.0 to assess the model fit by four general indexes: TLI, CFI, RMSEA, and SRMR (Hu and Bentler, 1999). The admissible cutoff values were: greater than 0.90 for TLI and CFI and less than 0.08 for RMSEA and SRMR (Kline, 2011).

We used PROCESS macro (version 2.15) to test the hypothesized model with bootstrap methods. PROCESS was developed by Hayes (2013) and has been iteratively updated until 2016. Hayes (2015) suggests that the effect of a first-stage moderated mediation is precisely a linear function of the moderator; and the slope of this function is a product of the coefficient of the XW on M and the coefficient of M on Y.^1^ This product is also called an INDEX of the moderated mediation. If the INDEX is significantly different from zero, it indicates that the first-stage indirect effect is moderated. We used 5,000-sample bootstrapping in this study for all computations to output 95% bias-corrected confidence intervals. If the confidence interval excludes zero, it leads to the expectation that the indirect effect is linearly related to the moderator (Hayes, 2015).

## Results

Table 1 exhibits descriptive statistics and bivariate correlations of all the variables.

**Table 1:** Descriptive statistics and intercorrelations of variables.

	Mean	SD	1	2	3	4	5	6
1. Age	30.47	6.18	–					
2. Company tenure	4.42	4.10	0.64^**^	–				
3. Perceived ambidextrous organizational culture	3.85	0.69	−0.07	−0.07	**0.92**			
4. Psychological empowerment	4.06	0.62	−0.10^*^	−0.03	0.64^**^	**0.88**		
5. Innovative behavior	3.96	0.75	−0.04	−0.02	0.49^**^	0.58^**^	**0.86**	
6. Transformational leadership	4.16	0.55	−0.06	−0.06	0.70^**^	0.62^**^	0.48^**^	**0.95**

*n = 647. Internal consistency coefficients are reported in bold on the diagonal*.

* p < 0.05

** *p < 0.01*.

### Confirmatory Factor Analysis

We conducted a confirmatory factor analysis to validate the measures. Fit indexes suggested a good fit for our hypothesized four-factor model, with *χ*^2^ [49, *n* = 647] = 278.48, CFI = 0.97, TLI = 0.95, RMSEA = 0.08, and SRMR = 0.02. All of the observed items significantly loaded on expected latent factors. A mean loading of 0.84 indicated that convergent validity was acceptable. To further test our measures, we compared the hypothesized four-factor model to three alternative models: (1) a three-factor model with ambidextrous organizational culture and psychological empowerment loading on one latent factor, and the other constructs loading on their own respective factors, with Δ*χ*^2^ [3, *n* = 647] = 184.86, *p* < 0.01, which provided a worse fit than the hypothesized model; (2) a three-factor model with ambidextrous organizational culture and transformational leadership loading on one latent factor, and other variables loading on their own factors, with Δχ^2^ [3, *n* = 647] = 183.93, *p* < 0.01, which provided a worse fit than the hypothesized model; and (3) a two-factor model with employee-rated variables loading on one factor, and supervisor-rated outcome loading on another, with Δ*χ*^2^ [5, *n* = 647] = 1092.27, *p* < 0.01, which provided a worse fit than the hypothesized model. The results provide support for distinctiveness of the four constructs as hypothesized.

### The Mediating Role of Psychological Empowerment

Table 2 presents the result of the mediating effect. The total effect of ambidextrous organizational culture on innovative behavior is significantly positive (*b* = 0.39, *p* < 0.01). Table 2 also shows the direct effect of ambidextrous organizational culture on innovative behavior that excludes the indirect effect of the mediator.

**Table 2:** The regression analysis of mediating effect.

Effect	*B*	SE
Direct effect of X on M	0.57^**^	0.03
Direct effect of M on Y	0.40^**^	0.04
Total effect of X on Y	0.39^**^	0.03
Direct effect of X on Y	0.16^**^	0.03

*n = 647. All coefficients reported are unstandardized. X, Y, and M refer to independent variable, dependent variable, and mediator, respectively*.

** *p < 0.01*.

Furthermore, we adopted bootstrap methods to test the mediating role of psychological empowerment by SPSS PROCESS macro (version 2.15), which takes indirect effect into consideration (Shrout and Bolger, 2002). The mediating effect was tested with the expectation that the indirect effect should be non-zero (MacKinnon et al., 1995). The result shows that the indirect effect of ambidextrous organizational culture on innovative behavior *via* psychological empowerment was 0.23 (95% CI [0.1837, 0.2851]). The model fit of the mediating effect was acceptable (*R*^2^ = 0.40, *F*(1, 645) = 437.69, *p* < 0.01). With the confidence interval excluding zero, Hypothesis 1 is supported. We also tested the indirect effect of organizational diversity (0.21 with 95% CI [0.1626, 0.2620]) and shared vision (0.19 with 95% CI [0.1493, 0.2267]) on innovative behavior *via* psychological empowerment.

### The Moderating Role of Transformational Leadership

The interaction between ambidextrous organizational culture and transformational leadership in the first stage was tested. The result shows that transformational leadership significantly moderates the relationship between ambidextrous organizational culture and psychological empowerment (*b* = 0.15, *p* < 0.01), with an acceptable model fit (*R*^2^ = 0.49, *F*(3, 643) = 206.08, *p* < 0.01), and thus, Hypothesis 2 is supported. We also tested the moderating effects of transformational leadership on the effects of organizational diversity (*b* = 0.13, *p* < 0.01) and shared vision (*b* = 0.14, *p* < 0.01) on psychological empowerment, respectively. Furthermore, the conditional effect of perceived ambidextrous organizational culture on psychological empowerment at different values of the moderator (−1 SD as Low; +1 SD as High) is shown in Table 3. Moreover, considering that the moderator is a continuous variable, we used Johnson-Neyman technique (Preacher et al., 2007) to calculate the significance regions. The result shows that when transformational leadership is greater than 2.19 on a 5-point scale (perceived ambidextrous organizational culture as the independent variable), the direct effect is significantly different from zero. We further tested the two dimensions of perceived ambidextrous organizational culture: (1) when transformational leadership is greater than 2.09, the direct effect of organizational diversity on psychological empowerment is significantly different from zero. (2) When the value of transformational leadership varies from 1.79 to 3.08, the direct effect of shared vision on psychological empowerment is significantly different from zero.

**Table 3:** The conditional effect of perceived ambidextrous organizational culture on psychological empowerment.

Moderator	Effect	SE	LLCI	ULCI
Low	0.26	0.04	0.1841	0.3373
Mean	0.37	0.04	0.3042	0.4428
High	0.49	0.04	0.4052	0.5674

The conditional indirect effect of ambidextrous organizational culture on innovative behavior was computed by PROCESS, as shown in Table 4. The conditional indirect effect varied with different levels of the moderator (−1 SD as Low; +1 SD as High). All of the confidence intervals exclude zero, indicating that the conditional effects are significant.

**Table 4:** The conditional indirect effect of perceived ambidextrous organizational culture on innovative behavior through psychological empowerment.

Moderator	Effect	SE	LLCI	ULCI
Low	0.10	0.03	0.0570	0.1565
Mean	0.15	0.02	0.1084	0.2018
High	0.19	0.03	0.1446	0.2617

**Figure 2:**
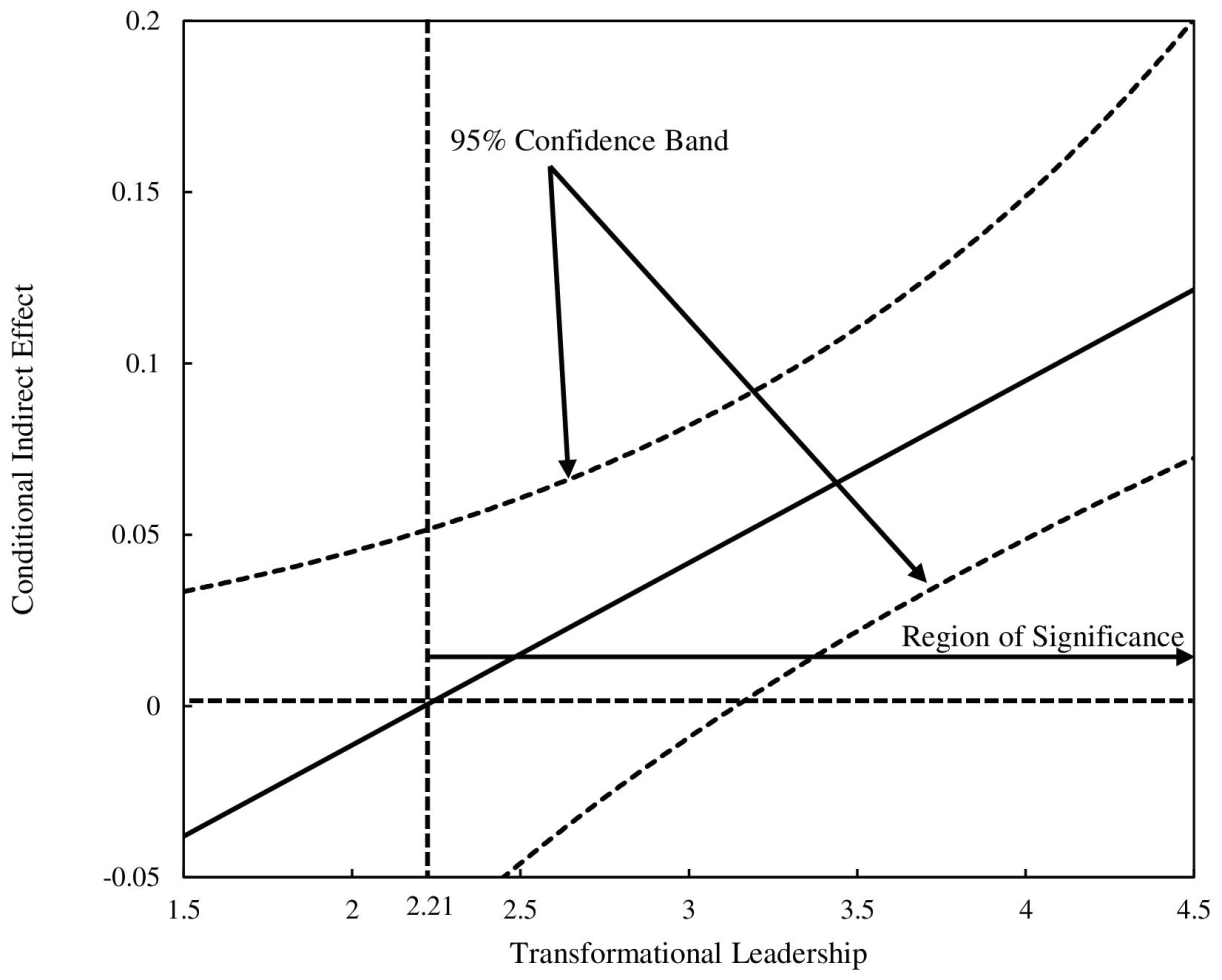
Johnson-Neyman regions of significance for the conditional effect of ambidextrous organizational culture at values of transformational leadership.

The INDEX of moderated mediation model, computed by PROCESS, was 0.06 (95% CI [0.1446, 0.2617]), with an acceptable model fit (*R*^2^ = 0.36, *F*(2, 644) = 183.83, *p* < 0.01), and thus, Hypothesis 3 is supported. We also tested the INDEX of organizational diversity (0.05 with 95% CI [0.0213, 0.0933]) and the INDEX of shared vision (0.07 with 95% CI [0.0347, 0.1010]). Given that the moderator (transformational leadership) is a continuous variable, we also used the Johnson-Neyman technique (Bauer and Curran, 2005; Preacher et al., 2007; Hayes and Matthes, 2009), which can provide a more detailed image of the conditional effect *via* the mediator, rather than the more common “pick-a-point” approach (Pollack et al., 2012). Using this technique, we obtained the “region of significance” for the conditional effect, referring to the range of the moderator in which the indirect effect is statistically different from zero. In Figure 2, the vertical dotted line represents the boundaries of the region of significance, and the pair of dotted curves represent 95% confidence band. As can be seen, when transformational leadership is greater than 2.21 (on a 5-point scale), the indirect effect is significantly different from zero, which indicates the effect of ambidextrous organizational culture on innovative behavior *via* psychological empowerment is significant and positively moderated by transformational leadership.

## Discussion

### Theoretical Implications

This study extends our knowledge of employees’ reactions related to innovation toward ambidextrous organizational culture, its underlying mechanism and conditional limit, making contributions to the psychology of organizational culture and individual innovation in three notable ways.

First, this study explores ambidextrous organizational culture from an individual perspective, and connects it with employees’ innovative behavior, which reveals the psychology of individual innovation within an organization. Previous studies mainly take organizational culture as a higher-order construct (e.g., Ogbonna and Harris, 2000; Jones et al., 2005), and take its development as a top-down process (Wang and Rafiq, 2014). The concept of ambidextrous organizational culture emphasizes employees’ involvement and participation, which is aligned with viewpoint that organizational innovation is achieved by innovative behavior of employees (Amabile, 1988). Employees embedded in an organization are influenced by organizational culture and simultaneously react upon the organization through their behavior (Meyer et al., 2010). The results of this study can provide a better understanding of employees’ impacts on organizational culture development and innovation from an individual perspective. Moreover, employees’ perceptions of organizational culture are important and have drawn scholars’ attention in recent years (e.g., Joo, 2010; Vijayakumar and Padma, 2014; Reis et al., 2016). The results of this study also indicate that how employees perceive organizational culture is more predictive of employees’ behavior than the culture itself, especially given that our data were collected from one company but employees’ perceptions still varied.

Second, this study combines ambidextrous organizational culture and employees’ innovative behavior by the intermediate effect of psychological empowerment. Ambidextrous organizational culture is thought as a pro-innovation factor (Wang and Rafiq, 2014), and its underlying mechanism approaching individual innovation mainly focuses on a resource-based view (Gibson and Birkinshaw, 2004). This study use self-determination theory to build a linkage that relates ambidexterity grounded in organizational culture to individual innovation *via* psychological empowerment, revealing that employees’ intrinsic motivation plays an important role in transforming organizational culture incorporating ambidexterity to individual innovation.

Specifically, based on the perspective of organizational ambidexterity, ambidextrous organizational culture has two dimensions: organizational diversity, and shared vision. Organizational diversity encourages employees to think and behave originally and autonomously, which helps not only exploration but also generating new ideas to improve extant systems or processes required by exploitation (Ahuja and Lampert, 2001). Meanwhile, shared vision helps organizational members to integrate their individual goals and actions with collective goals and actions (Leana and Van Buren, 1999), which translates different ideas into focused behavior required by exploitation (McGrath et al., 1994). Therefore, organizational diversity and shared vision combine together and reinforce each other to form ambidextrous organizational culture that helps integrate exploration and exploitation of ambidexterity.

Moreover, the results of this study reveal that the bottom-up process of ambidextrous organizational culture development involves employees and can be perceived by them. During this process, their psychological needs of autonomy, competence, and relatedness are fulfilled, and consequently they get psychologically empowered (Seibert et al., 2011; Fernandez and Moldogaziev, 2015) and exhibit innovative behavior in the workplace (Seibert et al., 2011). Our findings also extend the scope of self-determination theory applied in linking ambidextrous organizational culture and innovative behavior, implying that there is not only a resource-based approach but also a motivational approach with the mediating effect of psychological empowerment while exploring the relationship between ambidexterity and innovation.

Third, this study explores the impact of interaction of culture and leadership on innovative behavior. Previous studies find that organizational culture (see Hogan and Coote, 2014; Naranjo-Valencia et al., 2017) and leadership (see Pieterse et al., 2010; Aryee et al., 2012) are separately related to innovative behavior, and little attention has been given to dig into the impact of the association of these two concepts (Ogbonna and Harris, 2000). This study finds that the reinforcement to innovative behavior arises from the interaction of ambidextrous organizational culture and transformational leadership through psychological empowerment, which indicates that higher level factors (organizational context and leaders) jointly shape employees’ cognition and understandings of their work roles and thus their individual behavior related to innovation. Moreover, the results also suggest that applying a framework of self-determination theory, transformational leadership can be considered as a facilitator to the effect of ambidextrous organizational culture on innovative behavior through psychological empowerment, which provides us a new motivational perspective to investigate roles that transformational leaders play within an organization.

### Practical Implications

The findings of this study reveal several ways in which organizations can strategically focus on their culture and leadership efforts. First, organizations should make efforts to sustain the effects of ambidextrous organizational culture and engage in more investment in ambidextrous training which emphasizes creativity and discipline at the same time. Employees’ perceptions of their organization’s ambidextrous organizational culture will influence their psychological empowerment and innovative behavior at work, as the results show. This leads to our first suggestion that organizations should not only make sure they have developed values and beliefs that promote innovation but also communicate them to employees in an appropriate way. Since organizational culture can create an environment that provides clues for employees how to behave in specific contexts (Schein, 2004), organizations are advised to clarify the norms and principles that are encouraged and embedded in their culture. Additionally, it is intriguing that our data were collected from one company, but the level of employees’ perceptions of organizational culture varied significantly. We speculate that a large-scale enterprise has numerous bureaus and their understandings of organizational culture are different because of work roles, job requirements, and so on. Accordingly, it is vital that employers communicate shared vision and collective goals to their employees.

Moreover, the findings of this study provide evidence that diversity is beneficial to innovation, as well as psychological empowerment. Our findings suggest that organizations should focus more on activities that encourage employees to recognize and reward differences in skills, knowledge, and opinions, which are intangible and task-related compared with visible dissimilarity such as demographic heterogeneity (Cox, 1994; Rink and Ellemers, 2007). Specifically, employers should make efforts to enhance employees’ openness toward diversity in the work place rather than preventing or reducing dissimilarity related to tasks. Furthermore, shared vision helps members of an organization actively contribute diverse ideas and skills by encouraging activities such as knowledge transfer and resource exchange (Tsai and Ghoshal, 1998), which are prerequisite to apply useful information to collective goals (Wang and Rafiq, 2014). Hence, we recommend integrating diversity and shared vision within organizations in order to assure the benefits from ambidexterity. In addition, there is evidence showing that, compared with actual dissimilarity and diversity, perceived dissimilarity and diversity are more impactful on employees’ behavior (see Orpen, 1984; Turban and Jones, 1988; Strauss et al., 2001). Therefore, organizations are advised to focus more on fostering a positive and constructive climate for diversity, under which employees’ perceptions of diversity are more likely to lead to favorable outcomes.

Second, as transformational leadership is one of the most important stimulators of employees’ intelligence (Bass, 1990), organizations should express special concern about training in supervisors’ transformational behaviors. Leaders are the main entity that communicates organizational culture to their subordinates (Bass and Avolio, 1993). Meanwhile, the fit between leadership style and organizational culture is important for effectiveness of the organization (Bowers et al., 2017). Accordingly, as our findings show a mutual benefits of transformational leadership and ambidextrous organizational culture, organizations should inspire leaders to be charismatic, individually considerate, and intellectually stimulating (Bass, 1990), meaning that leaders should focus on behaviors such as instilling a sense of mission, encouraging employees to pay attention to higher-level concerns, and articulating a shared vision, beyond just exchanging material and resources.

Third, as psychological empowerment is not a personal trait that remains stable across situations, but rather a set of cognitive factors shaped and influenced by organizational contexts (Thomas and Velthouse, 1990), organizations are advised to establish environments that cultivate employees’ empowerment. More specifically, employers should not only focus on activities concerned with empowerment, but also provide environments in which employees can perceive self-efficacy, autonomy, and that what they do is impactful and meaningful.

### Limitations and Future Direction

This study has several limitations that future research could overcome. First, as this study focuses on perceived ambidextrous organizational culture and innovative behavior at the individual level, it would be helpful for future studies to explore whether the impacts of employees’ perceptions of ambidextrous organizational culture and actual culture are different. In addition, although our findings show that perceived ambidextrous organizational culture varies significantly and all of our hypotheses are supported, our data were collected from only one company. Future research could collect data from additional companies to compare innovative effectiveness in the presence of different levels of actual ambidextrous organizational culture.

Second, all of the items measured in this study reflect positive characteristics, which might result in a tendency of social desirability considering that respondents are likely to behave in a culturally acceptable manner (Thomas and Kilmann, 1975). Moreover, most of the items were measured by participants’ self-reported ratings. Despite the fact that self-reported measures are among the most valid and useful data sources for assessing individual perceptions and attitudes (Glick et al., 1986), this may lead to common method variance (Spector, 1994). Future research could consider the use of more objective measures.

Finally, we followed several procedural remedies to minimize common method variance (see Podsakoff et al., 2003), including measuring predictors and criteria from different sources, using temporal separation of measurement, and ensuring respondents’ confidentiality. Furthermore, we ran a one-factor model (see Harman, 1967) to make sure that common method bias should not nullify our findings, and its poor fit (*χ*^2^ [54, *n* = 647] = 2021.71, CFI = 0.71, TLI = 0.64, RMSEA = 0.24, and SRMR = 0.17) indicates that no single factor can explain a majority of the variance. Future research should also concern the problem of common method bias.

## Data Availability Statement

The raw data supporting the conclusions of this manuscript will be made available by the authors, without undue reservation, to any qualified researcher.

## Ethics Statement

This study was carried out in accordance with the recommendations of academic ethics guidelines of the insititutional review board of Ningbo Institute, Zhejiang University with written informed consent from all subjects. All subjects gave written informed consent in accordance with the Declaration of Helsinki. The protocol was approved by the insititutional review board of Ningbo Institute, Zhejiang University.

## Author Contributions

YL contributed to proposing core concepts and the theoretical model. WW (corresponding author) contributed to providing theoretical and methodological support and modifying the article. DC contributed to collecting data. All the authors made efforts to manuscript writing and revising.

### Conflict of Interest

DC was employed by company Hikvision Digital Technology Co. Ltd.

The remaining authors declare that the research was conducted in the absence of any commercial or financial relationships that could be construed as a potential conflict of interest.

## Footnotes

^1^X/Y means independent/dependent variable, while M/W refers to mediator/moderator, respectively, and hereinafter.

## Appendix: Key Constructs and Items

### Perceived Ambidextrous Organizational Culture

In this business unit,

1. we respect everyone’s different viewpoints (organizational diversity 1).
2. we value people from diverse backgrounds with diverse experiences and skills (organizational diversity 2).
3. we encourage all employees to generate as many alternative solutions to problems as possible (organizational diversity 3).
4. all of us view ourselves as partners in charting the direction of this business unit (shared vision 1).
5. the future direction of this business unit is clearly communicated to everyone (shared vision 2).
6. everyone who works here is well aware of the long-term plans and direction of this business unit (shared vision 3).
7. there is a strong sense of where this business unit is going (shared vision 4).

### Psychological Empowerment

1. The work I do is very important to me (meaning 1).
2. My job activities are personally meaningful to me (meaning 2).
3. The work I do is meaningful to me (meaning 3).
4. I am confident about my ability to do my job (competence 1).
5. I am self-assured about my capabilities to perform my work activities (competence 2).
6. I have mastered the skills necessary for my job (competence 3).
7. I have significant autonomy in determining how I do my job (self-determination 1).
8. I can decide on my own how to go about doing my work (self-determination 2).
9. I have considerable opportunity for independence and freedom in how I do my job (self-determination 3).
10. My impact on what happens in my department is large (impact 1).
11. I have a great deal of control over what happens in my department (impact 2).
12. I have significant influence over what happens in my department (impact 3).

### Innovative Behavior

Supervisor indicated how characteristic each of the following behaviors was of a particular employee:

1. Searches out new technologies, processes, techniques, and/or product ideas.
2. Generates creative ideas.
3. Promotes and champions ideas to others.
4. Investigates and secures funds needed to implement new ideas.
5. Develops adequate plans and schedules for the implementation of new ideas.
6. Is innovative.
